# Semapimod Sensitizes Glioblastoma Tumors to Ionizing Radiation by Targeting Microglia

**DOI:** 10.1371/journal.pone.0095885

**Published:** 2014-05-09

**Authors:** Ian S. Miller, Sebastien Didier, David W. Murray, Tia H. Turner, Magimairajan Issaivanan, Rosamaria Ruggieri, Yousef Al-Abed, Marc Symons

**Affiliations:** 1 Center for Oncology and Cell Biology, The Feinstein Institute for Medical Research at North Shore-LIJ, Manhasset, New York, United States of America; 2 Center for Molecular Innovation, The Feinstein Institute for Medical Research at North Shore-LIJ, Manhasset, New York, United States of America; University of Florida, United States of America

## Abstract

Glioblastoma is the most malignant and lethal form of astrocytoma, with patients having a median survival time of approximately 15 months with current therapeutic modalities. It is therefore important to identify novel therapeutics. There is mounting evidence that microglia (specialized brain-resident macrophages) play a significant role in the development and progression of glioblastoma tumors. In this paper we show that microglia, in addition to stimulating glioblastoma cell invasion, also promote glioblastoma cell proliferation and resistance to ionizing radiation *in vitro*. We found that semapimod, a drug that selectively interferes with the function of macrophages and microglia, potently inhibits microglia-stimulated GL261 invasion, without affecting serum-stimulated glioblastoma cell invasion. Semapimod also inhibits microglia-stimulated resistance of glioblastoma cells to radiation, but has no significant effect on microglia-stimulated glioblastoma cell proliferation. We also found that intracranially administered semapimod strongly increases the survival of GL261 tumor-bearing animals in combination with radiation, but has no significant benefit in the absence of radiation. In conclusion, our observations indicate that semapimod sensitizes glioblastoma tumors to ionizing radiation by targeting microglia and/or infiltrating macrophages.

## Introduction

Glioblastoma Multiforme (GBM) is the most malignant and lethal form of astrocytoma (WHO grade IV). Despite recent advancements in the standards of care, the outlook for GBM patients remains bleak and new therapies are therefore swiftly required. Currently, the standard of care is gross tumor resection followed by radiation treatment and concurrent chemotherapy [1]. However, even after extensive therapy, relapse is certain and the disease remains lethal, with a life expectancy of little over one year after diagnosis [2].

Microglia are the resident macrophages of the central nervous system (CNS) [3], [4]. They are glial cells that are of hematopoietic origin. Microglia are the main phagocytic and immunocompetent cells in the CNS. They are activated by damage or infection and phagocytose debris and other cells. They also present antigens and secrete cytokines that regulate inflammatory responses. Microglia are attracted by glial tumors via multiple tumor-secreted factors and are enriched in the tumor periphery [5], [6], [7]. The extent of microglia infiltration correlates with tumor grade [8], [9]. In glioblastoma, microglia account for as much as 30% of the tumor mass [10]. Instead of producing an anti-tumor effect, microglia are co-opted by the tumor to favor its growth [5], [6], [7]. In this reprogrammed state, they support the tumor by secreting factors that promote immunosuppression, and glioblastoma cell proliferation, invasion and angiogenesis. Importantly, selective ablation of microglia has been shown to inhibit glioblastoma invasiveness and growth [11], [12].

Semapimod (initially termed CNI-1493) is a multivalent guanylhydrazone small molecule that was developed as a targeted inhibitor of cytokine-inducible L-arginine transport in macrophages [13], [14]. Remarkably, no significant effect of semapimod on cells from other lineages has been noted [15]. Semapimod has been shown to have significant efficacy in several animal models of inflammation, including lethal sepsis [16]. Importantly, semapimod has already been tested in a phase II clinical trial for Crohn's disease and was shown to be very well tolerated in humans [17]. In this study, we examined whether semapimod can blunt the stimulatory effect of microglia on the malignant behavior of glioblastoma cells, both *in vitro* and in a syngeneic orthotopic mouse model of glioblastoma.

## Materials and Methods

### Ethics Statement

All procedures involving mice were conducted in accordance with the National Institutes of Health regulations and ARRIVE guidelines concerning the use and care of experimental animals. The study of mice was approved by the Institutional Animal Care and Use Committee (IACUC) of the Feinstein Institute. All surgeries were performed under isoflurane anesthesia, and all efforts were made to minimize suffering.

### Cell Culture

Murine GL261 glioblastoma cells were obtained from the National Cancer Institute (Frederick, MD, USA) and normal murine microglia, isolated from C57Bl/6 mice [18], were obtained from S. Coniglio (Albert Einstein College of Medicine) [19]. Both cultures were maintained in Macrophage Serum-Free Medium (MSFM; Life Technologies Corporation) with 10% fetal calf serum. Microglia were supplemented with 10 ng/mL recombinant mouse granulocyte macrophage–colony-stimulating factor (GM-CSF) (R&D Systems). All cultures were grown at 37°C in a humidified atmosphere of 5% CO_2_, 95% air. Cell lines were tested for the presence of contaminating mycoplasma during experimentation.

### Reagents

Semapimod was produced in house by Dr. Yousef Al-Abed and prepared as a stock concentration of 20 mM in 7% DMSO and ddH_2_0. It was diluted for experiments using Dulbecco's phosphate-buffered saline (PBS) to concentrations required.

### Invasion Assays

Glioblastoma and microglial cells were labelled with cell tracker green CMFDA and with cell tracker red CMTPX, respectively, and then embedded in 50 µL of 10 mg/mL basement membrane extract (BME) (Trevigen). The mixture was then placed in a transwell insert (previously coated with 1 µg/mL fibronectin on the bottom side of the 8 µm filter to maintain adhesion of the cells that invaded through the filter) and allowed to polymerize for 30 min at 37°C. Subsequently, serum free medium was added to both wells. To maintain constant cell numbers, cells were plated at a density per invasion chamber of 15×10^4^ GL261 cells and 5×10^4^ microglia cells in MSFM. Semapimod or its diluent was added at varying concentrations into the BME and in the media above and below the transwell. Invasion chambers were incubated for 48 h and subsequently fixed in 3.7% formaldehyde in phosphate-buffered saline (PBS). The gel in the transwell inserts was carefully removed. Invaded glioblastoma cells were imaged with a Zeiss Axiovision inverted microscope and a 10× objective. All invaded cells were counted.

To measure the invasion of microglia toward glioblastoma cells *in vitro*, we used a variant of the glioblastoma cell invasion assay. First, 15×10^4^ Gl261 cells were plated in MSFM medium overnight and subsequently were placed in serum free medium. Subsequently, 5×10^4^ microglia cells were embedded in 50 µL of 10 mg/mL BME with or without 200 nM semapimod, placed in a 8 µm transwell (previously coated with 1 µg/mL fibronectin on the bottom side of the 8 µm filter to maintain adhesion of the cells that invaded through the filter) and allowed to polymerize for 30 min at 37°C. The transwell chambers were then placed above the previously plated GL261. After 48 h of incubation, the chambers were fixed in 3.7% formaldehyde in PBS. The cells were stained with 0.2% crystal violet in 2% ethanol. The remaining BME was carefully removed and the inserts were allowed to dry. Cells attached to the bottom of the filter were imaged as for the invasion assay and the total number of invaded microglia was determined.

### Colony Formation Assay

Gl261 cells and microglia were labeled as described for the invasion assay and were co-cultured in serum overnight and then changed to serum free medium for 48 h in the absence of serum at a 1∶1 ratio at a density of 5×10^5^ per well of a 6-well plate in the presence or absence of 200 nM semapimod. Subsequently, cells were subjected to 3 Gy of X-ray irradiation using a Radionics 160 kV irradiator. Two days after irradiation, cells were trypsinized, counted using a fluorescence microscope and 500 GL261 cells were plated in 6 cm dishes containing 10% FBS MSFM medium. Colony formation was allowed to proceed for 12 days, with medium changes every other day. Subsequently, the cells were fixed in 3.7% formaldehyde in PBS and stained with 0.2% (w/v) sulpharhodamine B (SRB) dye in 1% acetic acid for 20 min. The dishes were washed with 1% glacial acetic acid and allowed to dry. Plates were scanned and processed using Photoshop 5 (Adobe). Images were changed to grayscale and background threshold intensity was set. Colonies were counted automatically by Image J (*rsbweb.nih.gov/ij/*) and the numbers graphed.

### Proliferation Assay

50,000 GL261 cells were cultured overnight in the absence or presence or microglia (50,000 or 150,000) in serum-containing medium and subsequently cultured in the presence or absence of 200 nM semapimod in a 6-well dish in serum-free conditions. Microglia were added in a ratio of 1∶1 or 1∶3 GL261:microglia. After 72 h of incubation, plates were washed in PBS and fixed in 3.7% formaldehyde in PBS. In initial experiments, we stained the microglia with tomato lectin for 45 mins and stained all nuclei with DAPI. Ten fluorescence micrographs of each culture were taken with a Zeiss Axiovert-based imaging system. Counting of the cells showed that the number of the microglia did not change over the observation period of 3 days. Therefore in subsequent experiments the cells were stained with SRB as described for the colony formation assay. After drying, the plates were de-stained with 500 µL of 10 mM Tris base (Sigma). Eluted SRB was measured by absorbance at 490 nm. To determine the cell growth, the OD at day 1 was subtracted from the OD at day 3.

### Animal Experiments

First, 8 week old male C57BL/6J mice were inoculated in the right caudate putamen with GL261 cells. Briefly, animals were placed in a stereotactic frame after pre-anesthesia exposure in a box of 5% isoflurane. Deep anesthesia was maintained on the frame at approximately 2% isoflurane and 2% O_2_. A burr hole was drilled 1 mm anterior and 2.5 mm to the right of the bregma. With the aid of the stereotactic frame, GL261 cells (2×10^4^ cells suspended in 1 µL) were injected with a Hamilton syringe at a depth of 3 mm over a one minute time period. Subsequently, the syringe was left in place for 1 minute to prevent reflux.

Seven days after implantation of the GL261 cells, an osmotic pump (Alzet, Durect) filled with either 0.42 mg/ml solution of semapimod or vehicle was implanted in a subcutaneous pocket on the dorsal flank of the animal. A catheter with attached cannula delivered the drug intracranially over a period of 2 weeks, at a steady rate of 0.25 µl/h, which is equivalent to a dose of 6 mg/kg/day, assuming no blood-brain barrier permeability of the drug. Starting one day after pump implantation, animals were given 2 Gy whole brain irradiation every other day over a period of 10 days (10 Gy total). Animals that lost greater than 20% in body weight or displayed paralysis and/or lack of grooming, were deemed moribund and euthanized immediately.

Brains from tumor-bearing mice were frozen on dry ice and stored at −80°C until processing. The brain was sliced using a cryostat at −20°C at a thickness of 40 µm (for tumor sizing) or 8 µm for immunohistochemistry. Slices were placed on pre-coated poly-l-lysine slides (Superfrost, Fisher) and fixed in 3.7% formaldehyde in PBS. Sections were stained with Harris-modified haematoxilin solution. Activated microglia were visualized with an Iba1 antibody (Wako). Tumor cell invasion was determined by counting the number of Ki67 (Millipore) positive nuclei located beyond the tumor border. The border was outlined by determining where the bulk of the tumor material was found by Ki67 staining. Tumor size was determined as the area of the largest cross section of the tumor, multiplied by the depth of the tumor.

The cell density of the tumor was calculated by counting the total number of cells in 10 fields taken over different sections and multiplying the tumor area by the thickness of the slice (8 µm) to obtain total number of cells in a tumor section. This was then divided by the volume of the tumor section to obtain the number of cells per unit volume. To obtain the total number of cells in a tumor, the cell density was multiplied by the calculated volume of the tumor.

### TUNEL Assay

Frozen brains were sectioned and tested for apoptosis using the TACS 2 TdT-DAB in Situ Apoptosis Detection Kit from Trevigen following the manufacturer's recommendations. Slices were imaged with the Zeiss Axiovert 200 M microscope and a 40× objective. Images were analyzed with the Axiovision program. For each tumor, 20 to 60 fields were analyzed.

### Statistics

For the experiments determining the therapeutic benefit of semapimod in animals, a chi-squared test with 3 degrees of freedom was used. For all other experiments a two-tailed Student's *t*-test was used.

## Results

### Semapimod inhibits microglia-stimulated glioblastoma cell invasion

In order to examine the effect of microglia on the malignant properties of glioblastoma cells, we used GL261 murine glioblastoma cells and syngeneic microglia isolated from C57Bl/6 mice, a model system that has been extensively used to study the reciprocal interaction between glioblastoma cells and microglia [11], [12], [19].

**Figure 1:**
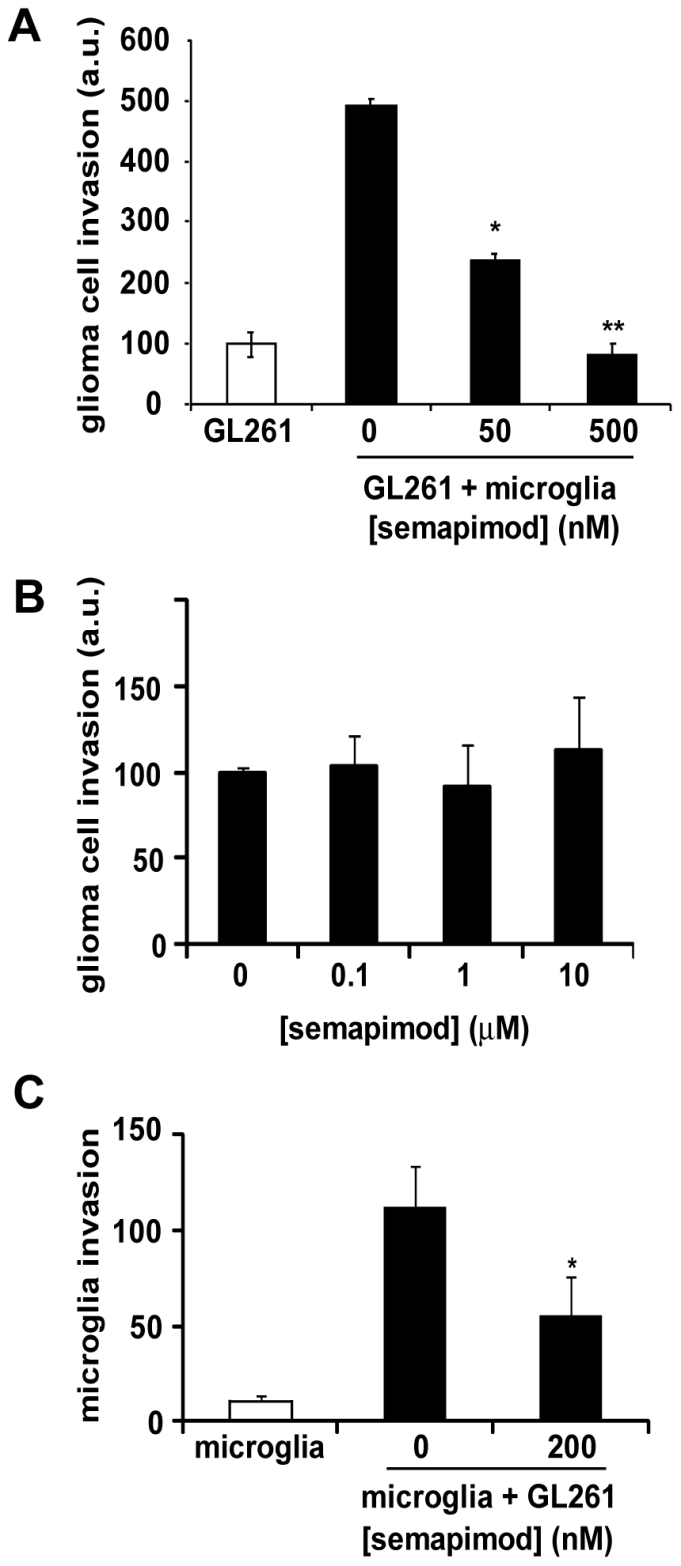
Semapimod inhibits microglia-stimulated glioblastoma invasion *in vitro*. (A) Semapimod inhibits microglia-stimulated glioblastoma cell invasion. GL261 cells were embedded in basement membrane extract (BME) with or without microglia in the presence of the indicated concentrations of semapimod, layered in the transwell and incubated for 48 h. The total number of invading GL261 cells was determined and normalized to that of GL261 cells in monoculture. Data shown represent the average +/− SEM of 3 independent experiments, performed in duplicate. (B) Semapimod does not affect serum-stimulated glioblastoma cell invasion. GL261 cells embedded in BME in the presence of the indicated concentration of semapimod, were layered in the transwell and incubated for 48 h. Serum (10% FBS) was added to the bottom well. The total number of invading GL261 cells was determined and normalized to that of GL261 cells in the absence of drug. Data shown represent the average +/− SEM of 3 independent experiments. (C) Semapimod inhibits glioblastoma cell-stimulated microglia invasion. Glioblastoma cells were plated in the bottom well and microglia were embedded in BME in the presence or absence of 200 nM semapimod, layered in the transwell and incubated for 48 h. The total number of invading microglia cells was determined. Data shown represent the average +/− SEM of 3 independent experiments. *: p<0.05, **: p<0.01 student's 2 tailed t-test.

A defining feature of malignant glioblastoma is the diffuse invasion of tumor cells into the surrounding parenchyma and microglia have been shown to strongly promote this activity [11], [19]–[22]. To determine the effect of microglia on the invasive properties of glioblastoma cells, we designed a novel 3-dimensional microglia–glioblastoma co-culture assay that provides a better approximation of the *in vivo* setting than a 2-dimensional configuration. Fluorescently labeled cells were embedded in reconstituted extracellular matrix (BME) in the absence of added serum at a ratio of 3∶1 glioblastoma cells to microglia and subsequently placed in transwells provided with an 8 µm pore filter. Using this assay, we observed that microglia stimulate GL261 invasion up to 5 fold and that semapimod interferes with this effect with an IC_50_ of less than 50 nM (Fig. 1A), similar to the IC_50_ of semapimod in inflammatory cytokine release from macrophages [14]. Importantly, semapimod, even at a concentration of 10 µM, does not affect serum-stimulated GL261 invasion, underlining the selectivity of semapimod for cells from the monocytic lineage (Fig. 1B).

### Semapimod inhibits glioblastoma-induced microglia invasion in vitro

Microglia extensively infiltrate glial tumors [6], [8], [9]. To examine whether semapimod can also inhibit migration of microglia towards glioblastoma cells, we established an *in vitro* transwell invasion assay by measuring the number of microglia that invade through a 3-dimensional extracellular matrix toward glioblastoma cells. We observed that the presence of GL261 cells in the bottom well strongly stimulates microglia invasion, by approximately 12 fold (Fig. 1C). This stimulatory effect is abolished by semapimod, with an IC_50_ of approximately 60 nM.

### Semapimod inhibits microglia-stimulated glioblastoma cell survival

**Figure 2:**
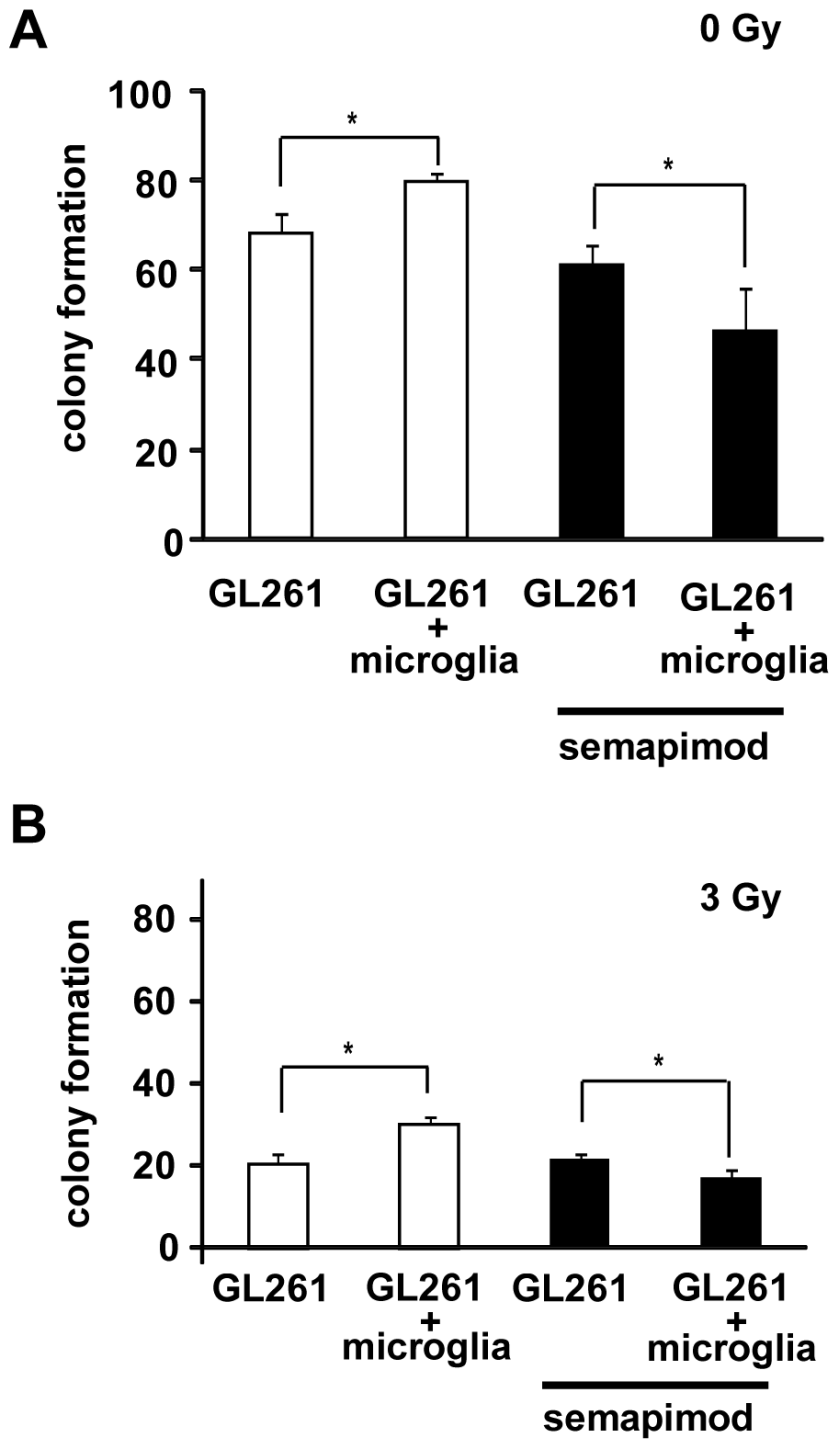
Semapimod removes microglia-induced radioprotection on GL261 *in vitro*. GL261 cells were cultured in the presence or absence of microglia and in the presence or absence of 200 nM of semapimod, followed by a colony formation assay. (A) Colony formation assay of GL261 after treatment with microglia and semapimod. (B) Determination of survival of GL261 after treatment with 3 Gy irradiation, microglia and semapimod. Data shown represent the average +/− SEM of 3 independent experiments. *: p<0.05 student's 2 tailed t-test.

A critical problem of malignant glioblastoma is its strong resistance to ionizing radiation (IR) and other therapeutic modalities [23]. The role of microglia in glioblastoma cell survival has not been studied thus far. We therefore examined whether microglia can enhance the survival of GL261 cells after IR and whether semapimod interferes with this function. GL261 cells were plated either in the presence or absence of microglia with or without semapimod (200 nM) for 2 days, followed by radiation treatment (3 Gy). Two days later, glioblastoma cell viability was assayed using a standard colony formation assay. We observed that co-culturing GL261 cells with microglia in the absence of radiation slightly, but significantly, stimulates their survival and that semapimod abolishes this (Fig. 2A). In the presence of radiation, the stimulatory effect of microglia on glioblastoma cell survival is more marked (50%) and this effect is also abolished by semapimod (Fig. 2B). In line with the selectivity of semapimod for cells from the monocytic lineage, semapimod does not affect the survival potential of glioblastoma cell monocultures. Thus, taken together, these data indicate that semapimod inhibits microglia-stimulated glioblastoma cell survival by modulating the activation state of the microglia.

### Semapimod does not affect microglia-stimulated glioblastoma cell proliferation

**Figure 3:**
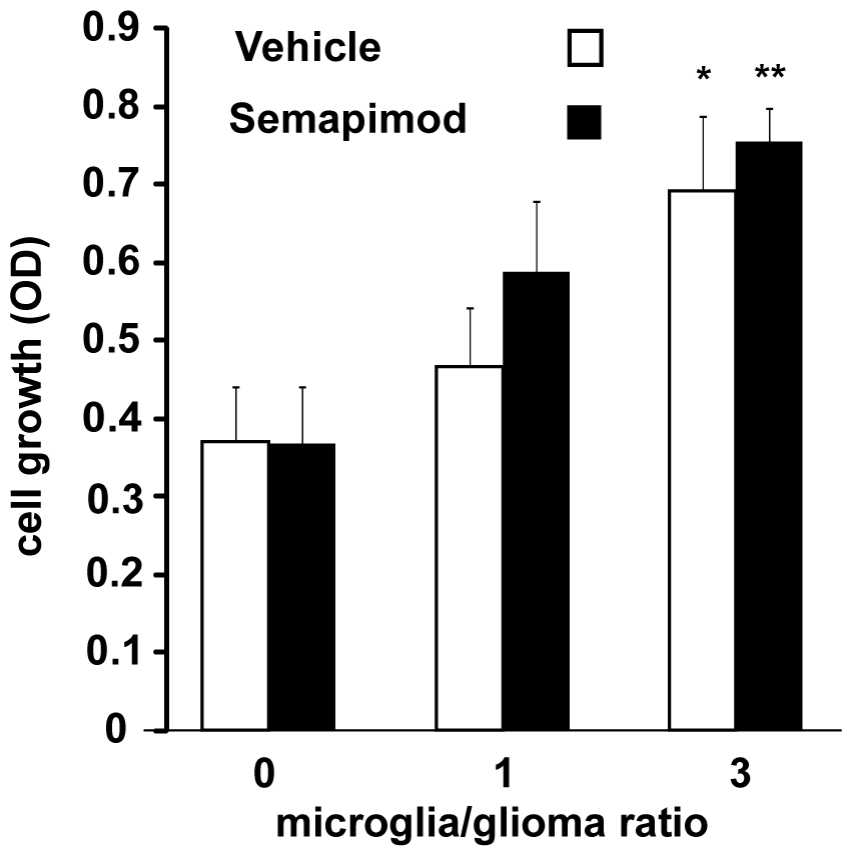
Semapimod does not affect microglia-stimulated growth of glioblastoma cells *in vitro*. GL261 cells were cultured in the presence or absence of microglia at the indicated ratio's, in the presence and absence of 200 nM semapimod. Cell growth over a period of 3 days was determined using the SRB method. Data shown represent the average +/− SEM of 3 independent experiments. *: p<0.05, **: p<0.01 student's 2 tailed t-test.

Microglia have been shown to slightly stimulate glioblastoma cell proliferation and therefore we also wanted to examine whether semapimod inhibits this effect. Fig. 3 shows that microglia stimulate GL261 cell proliferation in a fashion that depends on the microglia-glioblastoma cell ratio. However, semapimod (200 nM) does not affect the microglia-stimulated glioblastoma cell proliferation.

### Semapimod strongly inhibits tumor cell invasion in vivo

To evaluate the effect of semapimod on the malignant behavior of glioblastoma *in vivo*, we used orthotopic implantation of GL261 cells into syngeneic C57Bl/6 mice. This model displays all the pathological hallmarks of glioblastoma and is often used for examining the role of microglia in glioblastomagenesis and for pre-clinical evaluation of immunomodulatory therapies [11], [12], [19], [24].

**Figure 4:**
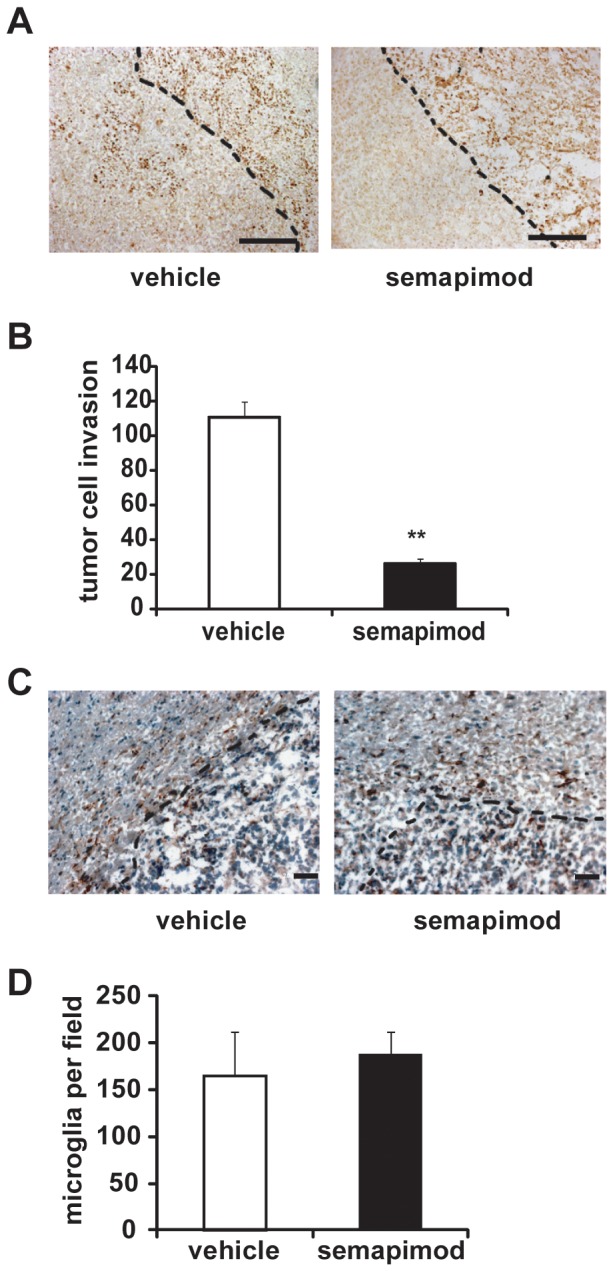
Semapimod inhibits glioblastoma cell invasion *in vivo*. GL261 cells were orthotopically implanted into C57Bl/6 mice. Starting 7 days after cell inoculation, the mice were treated intracranially for 1 week with semapimod, delivered via an osmotic pump. (A) Micrographs of tumor sections illustrating inhibition of GL261 cell invasion by semapimod. GL261 cells were visualized using Ki67 staining. The tumor borders are outlined. Scale bar represents 200 µm. (B) Quantification of the number of invaded GL261 cells normalized to the length of the tumor border (expressed in mm). Data shown represent the average +/− SEM of 5 different tumors. **: p<0.01 student's 2 tailed t-test. (C) Micrographs illustrating infiltration of microglia into GL261 tumors. Activated microglia were visualized using Iba1 staining. (D) Quantification of Iba1^+^ microglia infiltrated into the tumor. Scale bar represents 100 µm. Data shown represent the average +/− SEM of 5 different tumors.

In order to identify an effective dose of semapimod, we performed pilot studies and chose the minimal concentration that maximally inhibited tumor invasiveness one week after start of treatment. Animals were inoculated with 2×10^4^ GL261 cells into the right caudate putamen. To deliver semapimod, we used an osmotic pump that was implanted subcutaneously in the dorsal flank of the animal and fed a transcranial cannula that was inserted into the tumor. In order to mimic a therapeutic setting, tumors were allowed to develop for 1 week before the onset of treatment. To score tumor cell invasion, brain sections were probed for the presence of proliferative antigen Ki67. In line with our *in vitro* observations, semapimod, at a dose that is equivalent to 6 mg/kg/day, strongly inhibited tumor invasion. Whereas control tumors display diffuse edges and extensive tumor cell invasion into the surrounding parenchyma, semapimod-treated tumors have a sharply demarcated border (Fig. 4A). Quantification of the invading tumor cells revealed that semapimod inhibits tumor cell invasion by more than 75% (Fig. 4B).

As our *in vitro* observations showed that semapimod strongly inhibits invasion of microglia toward GL261 cells, we also examined tumor sections for the infiltration of microglia using Iba1 staining (Fig. 4C). No significant difference in the number of microglia per tumor area could be detected, however (Fig. 4D). The most likely interpretation for this finding is that semapimod treatment was started 7 days after the implantation of the tumor cells, a time at which microglia infiltration already has occurred [25].

**Figure 5:**
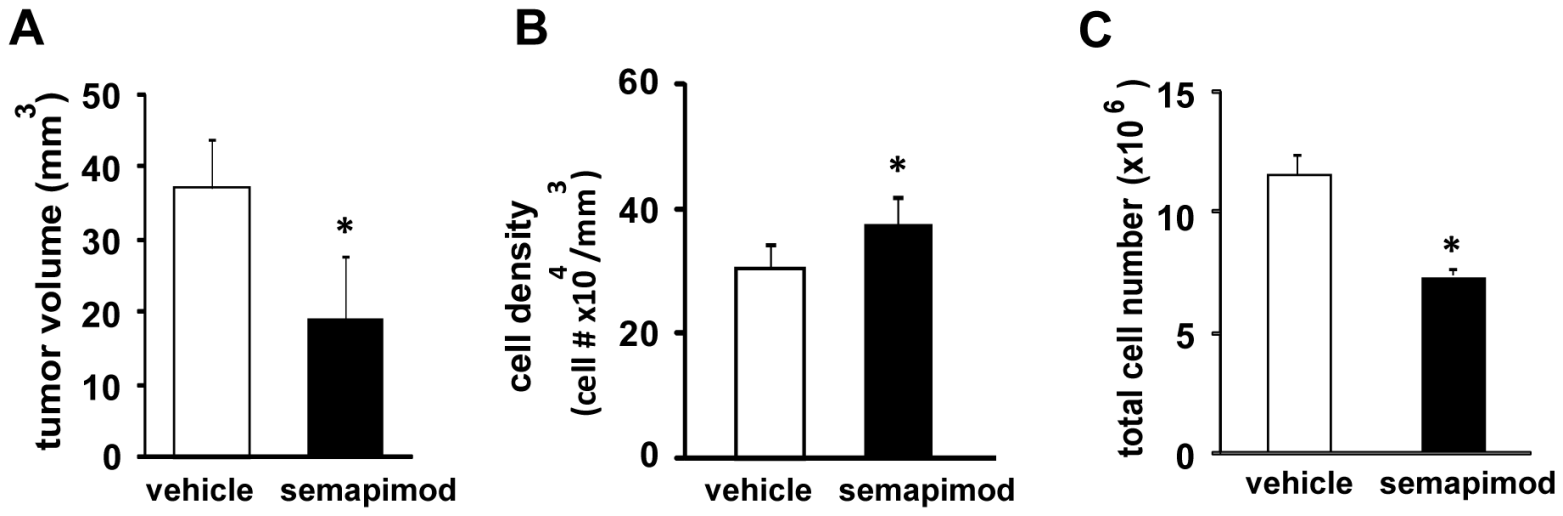
Semapimod inhibits tumor growth *in vivo*. GL261 cells were implanted and mice were treated as described in Fig. 4. (A) Quantification of tumor volume. Data shown represent the average +/− SEM of 5 different tumors. (B) Quantification of tumor cell density. Data shown represent the average +/− SEM of 5 different tumors. (C) Quantification of total tumor cell number. Data shown represent the average +/− SEM of 5 different tumors. *: p<0.05 student's 2 tailed t-test.

As semapimod does not affect GL261 cell proliferation *in vitro*, we were surprised by the finding that semapimod treatment causes a robust inhibition of tumor size, two weeks after tumor cell inoculation (Fig. 5A). Interestingly, the treated tumors also display a small but significant increase in tumor cell density (Fig. 5B), possibly caused by the strong inhibitory effect of semapimod on tumor cell invasion. Still, the estimated total number of cells in semapimod-treated tumors is significantly less than that in control tumors, indicating that semapimod indeed diminishes the proliferation potential of the tumor cells *in vivo* (Fig. 5C). The discrepancy between the *in vitro* and the *in vivo* tumor cell proliferation data may be due to the fact that the *in vivo* observations reflect the cumulative effect of many cell divisions, thereby possibly amplifying small differences in cell proliferation that are not detectable in the *in vitro* experiments.

### Semapimod strongly enhances the therapeutic efficacy of ionizing radiation in a syngeneic orthotopic mouse model of glioblastoma

**Figure 6:**
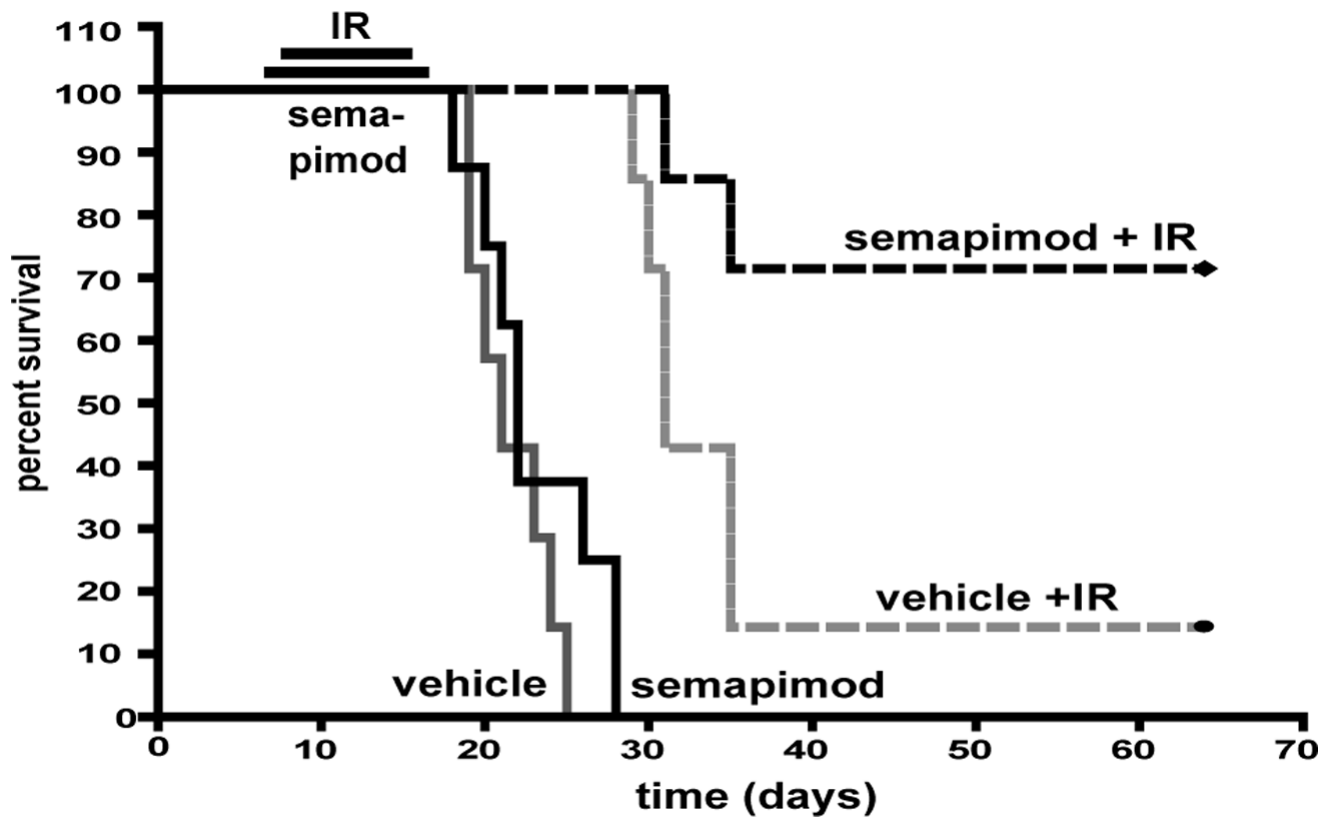
Semapimod increases survival of glioblastoma-bearing mice in conjunction with ionizing radiation. C57Bl/6 mice were injected orthotopically with GL261 cells. Starting 7 days after cell inoculation, the mice were treated intracranially for 2 weeks with semapimod, delivered via an osmotic pump. Starting on day 8, animals were given 2 Gy whole brain irradiation every other day over a period of 10 days (10 Gy total). *: p<0.05. Chi square test, with 3 degrees of freedom.

To determine the effect of semapimod on the survival of glioblastoma-bearing mice and to examine the effect of semapimod on radiation resistance *in vivo*, we randomized 4 groups of animals to receive semapimod or diluent in the absence or presence of 10 Gy fractionated whole brain irradiation. We observed that in the absence of radiation, semapimod does not significantly prolong survival (median survival of 22 days for semapimod-treated animals versus 20 days for controls) (Fig. 6). However, whereas radiation alone increased median survival by 12 days, most of the irradiated animals that were treated with semapimod survived at least 40 days beyond the median survival time of control animals and had no detectable tumors as judged by histological analysis (data not shown). These observations indicate that semapimod strongly sensitizes GL261 tumors to radiation.

**Figure 7:**
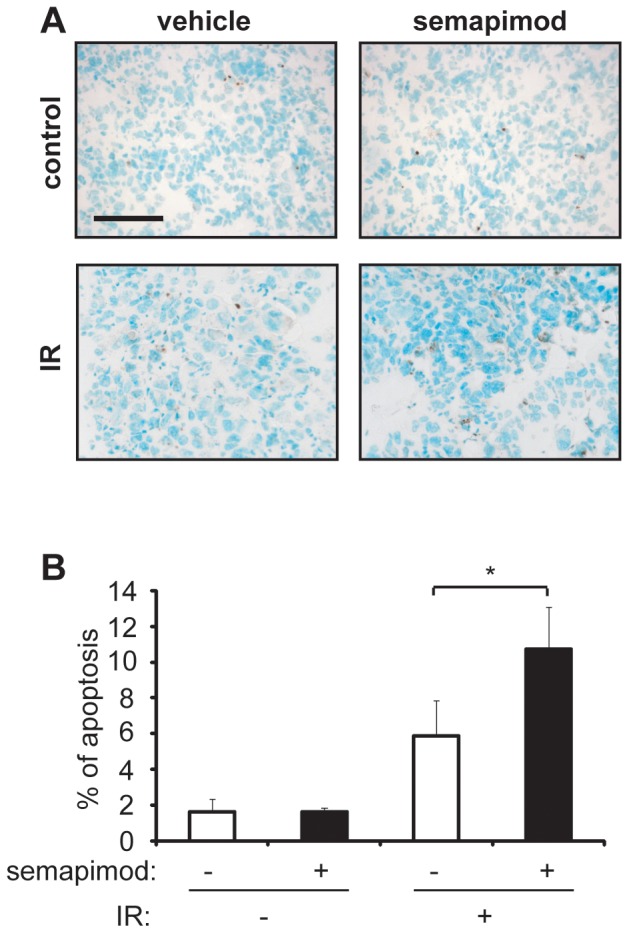
Semapimod increases apoptosis induced by radiation *in vivo*. GL261 tumors were generated in mice and treatments were carried out as described in Fig. 6, except that the total radiation was 8 Gy. The mice were euthanized the day after the last radiation treatment. Frozen brain sections were analyzed for apoptosis using TUNEL staining as described in Materials and Methods. (A) Micrographs of tumor sections illustrating TUNEL staining. (B) Quantification of TUNEL staining. The percentage of apoptotic cells was calculated using 20–60 micrographs per tumor section. Data shown represent the average +/− SEM of 3–4 different tumors. *: p<0.05 student's 2 tailed t-test.

To examine whether semapimod promotes radiotherapy-induced glioma cell death *in vivo*, we performed TUNEL staining on sections from control and treated tumors. Treatment with semapimod approximately doubled the percentage of apoptotic tumor cells in irradiated but not in non-irradiated animals (Fig. 7), strongly supporting the hypothesis that semapimod acts as a radiosensitizer in glioma tumors *in vivo*.

## Discussion

In this paper, we show that the immunomodulatory drug semapimod markedly enhances the survival of glioblastoma-bearing mice in conjunction with radiation therapy, but not as monotherapy. In addition, our *in vitro* observations show that semapimod inhibits the survival of irradiated glioblastoma cells in the presence, but not in the absence, of microglia. Taken together therefore, our results strongly suggest that semapimod sensitizes glioblastoma tumors to ionizing radiation by targeting microglia.

The notion that semapimod targets microglia, but not glioblastoma cells, is in line with previous data showing that semapimod is selective for cells derived from the monocytic lineage, i.e. microglia, macrophages and dendritic cells, but not T cells [15], [26]. We note that gliomas also are infiltrated by peripheral macrophages [25], which also may promote therapeutic resistance. Thus, the inhibitory effect of semapimod on the activation status of infiltrating macrophages may also contribute to the therapeutic effect of semapimod.

The mechanism of action of semapimod remains to be elucidated. The only known molecular target of semapimod to date is c-Raf [27]. However, we found that ERK activation in microglia that are stimulated by GL261-conditioned medium is not inhibited by semapimod (data not shown), suggesting that this proposed mechanism is not relevant for our observations. Exactly how semapimod modulates microglial function is currently under investigation in our laboratory.

We note that semapimod has been extensively used as an anti-inflammatory agent [27], [28]. Thus, our results that semapimod sensitizes tumors to radiation therapy may seem paradoxical, as the immune system, in general, is thought to counteract tumor formation. We note, however, that there is now mounting evidence in the literature that targeting microglia/macrophages may be of therapeutic benefit in the treatment of glioblastoma [5], [6], [7], [11], [12], [22], [25], [29] and other malignancies [30].

Hitherto, the role of microglia in the malignant behavior of glioblastoma cells has largely focused on the invasiveness of the tumor cells [5], [6] and the effect of microglia on the survival properties of glioblastoma cells has not been investigated. In this paper, we report that microglia exert a small, but significant, stimulatory effect on the survival of glioblastoma cells that are challenged by ionizing radiation and that semapimod inhibits this effect. In line with this result, we find that semapimod treatment increases the number of apoptotic tumor cells induced by ionizing radiation *in vivo* and markedly extends the survival of glioblastoma-bearing animals that are treated with radiation. The relatively large effect size of semapimod treatment observed *in vivo* may be explained by the use of a fractionated radiation regimen (5 doses of 2 Gy, every other day), which is expected to magnify the survival benefit seen in the single dose of irradiation in the *in vitro* setting.

The striking enhancement by semapimod of the therapeutic effect of ionizing radiation in glioblastoma-bearing mice is in line with the radiosensitization observed by depleting macrophages in a subcutaneous melanoma model [31] and the marked enhancement of the inhibitory effect of paclitaxel on mammary tumor pulmonary metastasis caused by a CSF-1R inhibitor that blocks macrophage recruitment to the tumor [32]. Notably, in all these cases, targeting the tumor-associated microglia/macrophage compartment on its own showed little or no therapeutic benefit. These observations strongly suggest that the microglia/macrophage compartment plays a critical role in therapeutic resistance and that targeting this compartment in combination with other therapeutic modalities is likely to be of significant clinical benefit.

We found that semapimod has a marked inhibitory effect on glioblastoma tumor cell invasion both *in vitro* and *in vivo*. These observations are in line with previous reports that have demonstrated that interference with the function of microglia or depletion of the microglial compartment has a strong inhibitory effect on glioblastoma cell invasion [11], [19]. Interestingly however, the marked inhibition in tumor invasiveness caused by semapimod is not accompanied by a significant increase in survival of glioblastoma-bearing mice. This observation suggests that blocking invasion on its own may have little therapeutic benefit, at least in the setting that we have explored. However, in other preclinical settings, inhibiting invasion could yield therapeutic benefit. Possible scenarios include the implementation of a tumor resection model [33], which better mimics current therapeutic approaches in the clinic, or combined treatment with bevacizumab, which has been shown to enhance the invasive behavior of glioblastoma tumors [34], [35].

An attractive feature of semapimod is that it is an investigational drug that already has been tested in several clinical trials [17], [36], [37]. Importantly, semapimod was shown to be very well tolerated in humans and displays a good safety profile. Thus, our observations suggest the utility of repositioning semapimod as an immunomodulator for the treatment of glioblastoma, as up-front adjuvant to standard therapy, concurrent with radiation and temozolomide chemotherapy [1].
